# Differential Gene Expression and Adherence of *Escherichia coli* O157:H7 *In Vitro* and in Ligated Pig Intestines

**DOI:** 10.1371/journal.pone.0017424

**Published:** 2011-02-28

**Authors:** Xianhua Yin, Jing Zhu, Yanni Feng, James R. Chambers, Joshua Gong, Carlton L. Gyles

**Affiliations:** 1 Guelph Food Research Center, Agriculture and Agri-Food Canada, Guelph, Ontario, Canada; 2 College of Life Science and Biotechnology, Shanghai Jiao Tong University, Shanghai, People's Republic of China; 3 College of Animal Science and Technology, Qingdao Agricultural University, Qingdao, People's Republic of China; 4 Department of Pathobiology, Ontario Veterinary College, University of Guelph, Guelph, Ontario, Canada; Institut de Pharmacologie et de Biologie Structurale, France

## Abstract

**Background:**

*Escherichia coli* O157:H7 strain 86–24 grown in MacConkey broth (MB) shows almost no adherence to cultured epithelial cells but adheres well in pig ligated intestines. This study investigated the mechanisms associated with the difference between *in-vitro* and *in-vivo* adherence of the MB culture.

**Methodology/Principal Findings:**

It was found that decreased adherence *in vitro* by bacteria grown in MB was mainly due to lactose, possibly implicating the involvement of carbon catabolite repression (CCR). Expression of selected virulence-related genes associated with adherence and CCR was then examined by quantitative PCR. When bacteria were grown in MB and Brain Heart Infusion with NaHCO_3_ (BHIN) plus lactose, pH was reduced to 5.5–5.9 and there was a significant decrease in expression of the locus of enterocyte effacement (LEE) genes *eae*, *tir*, *espD*, *grlA*/R and *ler*, and an increase in *cya* (cAMP), and two negative regulators of the LEE, *gadE* and *hfq*. Putative virulence genes *stcE*, *hlyA*, *ent* and *nleA* were also decreased *in vitro*. Reversal of these changes was noted for bacteria recovered from the intestine, where transcripts for *qseF* and *fis* and putative virulence factors AidA_15_, TerC and Ent/EspL2 were significantly increased, and transcripts for AIDA_48_, Iha, UreC, Efa1A, Efa1B, ToxB, EhxA, StcE, NleA and NleB were expressed at high levels.

**Conclusions/Significance:**

Presence of lactose resulted in decreased expression of LEE genes and the failure of EHEC O157:H7 to adhere to epithelial cells *in vitro* but this repression was overcome *in vivo*. CCR and/or acidic pH may have played a role in repression of the LEE genes. Bacterial pathogens need to integrate their nutritional metabolism with expression of virulence genes but little is known of how this is done in *E. coli* O157:H7. This study indicates one aspect of the subject that should be investigated further.

## Introduction

Enterohemorrhagic *Escherichia coli* (EHEC), represented by the protoserotype O157:H7, can colonize the intestine of humans and cause diarrhea, hemorrhagic colitis (HC) and hemolytic-uremic syndrome (HUS) [1]. One characteristic of EHEC O157 pathogenesis is the formation of attaching and effacing (AE) lesions, resulting in localized destruction of microvilli, cytoskeleton rearrangement and formation of pedestal-like structures underneath the bacteria, and leading to intimate adherence to and colonization of host intestine [2]. Formation of the AE lesion requires genes encoded on a pathogenicity island named the locus of enterocyte effacement (LEE), which is organized into five major operons: LEE1, LEE2, LEE3, tir/LEE5, and LEE4 [3], [4]. These operons encode a type III secretion system which secretes proteins involved in signal transduction and subversion of host cell functions, and the adhesin molecule intimin and its receptor (Tir) required for intimate host-cell interaction [5]. Another key virulence characteristic is the production of one or more verotoxins (VT), also called Shiga toxins (ST). VT is responsible for the tissue damage that leads to HC and HUS [6]. Severity of disease varies with the serotype of EHEC, with O157:H7 being the most prevalent and virulent serotype [6]. Many potential virulence factors have been found in various serotypes of EHEC and there is a correlation between the complement of putative virulence genes and association of the strains with severe disease and outbreaks [7], [8]. The potential virulence factors include chromosomally-encoded putative adhesins Efa1 (EHEC factor for adherence 1), Iha (IrgA homolog adhesin), and AIDA_15_ (the adhesin involved in diffuse adherence), tellurite resistance (Te^R^), urease, ent, NleA, NleB and NleD [6], [9], [10], [11], [12], [13], [14]. EHEC O157:H7 lacks a full length of *efa1*gene, but contains truncated version of *efa1* in the O-island (OI)-122, *efa1*'-a (Z4332) and *efa1*'-b (Z4333), whose expression and function in virulence has not been determined [15], [16].

EHEC O157:H7 possesses pO157, a non-conjugative plasmid that encodes several putative virulence factors including ToxB, EHEC hemolysin (Ehx), and a protease StcE [2], [17], [18]. Identification of the expression of these factors *in vivo* could clarify their roles in the virulence of this organism.

Colonization of the intestine is a key step in EHEC O157:H7 pathogenesis but this process is not completely understood. Further information on the adherence-related factors expressed *in vivo* is important to decipher EHEC adherence mechanisms. Factors involved in the colonization such as the LEE genes are regulated by a variety of environmental clues such as nutrient availability [19], via the actions of both global regulators and O157-specific regulators such as Hha, H-NS, IHF and rpoS [2], [20], [21], [22]. EivF and EtrA from type III secretion system 2 (ETT2) have been shown to strongly repress LEE gene expression [23]. EHEC also employs quorum sensing (QS) to control expression of its virulence genes [24]. The cyclic AMP (cAMP) receptor protein (CRP) is a major global regulatory protein in *E. coli* [25]. cAMP is a messenger signaling molecule whose intracellular level is modulated by environmental cues and carbon source [26], [27]. cAMP complexed with dimeric CRP has been studied extensively as a positive effector in carbon catabolite respression (CCR), in which the presence of glucose decreases the level of cAMP and represses the expression of enzymes involved in the metabolism of other carbon sources [28]. CCR is mainly mediated by the components of the phosphoenolpyruvate (PEP):carbohydrate phosphotransferase system (PTS), which include HPr (*ptsH*), EIIA^glc^ (*crr*), and EIIBC^glc^ (*ptsG*) [29]. Variation in the levels of the cAMP-CRP complex controls almost 200 operons in *E. coli* [30], [31].

Our previous study showed that adherence to tissue cultured cells by the bacteria grown in MacConkey broth (MB) was much less than that grown in BHI plus NaHCO_3_ (BHIN) [32]. One hypothesis that stemmed from this observation was that lactose and/or bile salts in MB might be responsible for this decreased adherence and CCR might be involved in the virulence gene regulation. *In vivo*, bacteria grown in MB caused similar levels of AE lesions to those by bacteria grown in BHIN. Bacterial behavior in the host is influenced not only by nutrient availability and carbon source, but also host factors, such as hormones and inflammatory agents, which affect production of virulence determinants [33], [34], [35], [36], [37]. The purposes of this study were: to investigate the effect of lactose and bile salts on bacterial adherence *in vitro*; to identify associated gene expression profiles of a set of ∼68 virulence-related factors; and to compare these results with the *in vivo* expressed genes from bacteria recovered from pig ligated intestine.

## Results

### Effects of lactose and bile salts on adherence of EHEC O157:H7 strain 86-24 to IPEC J2 and HEp-2 cells

**Figure 1:**
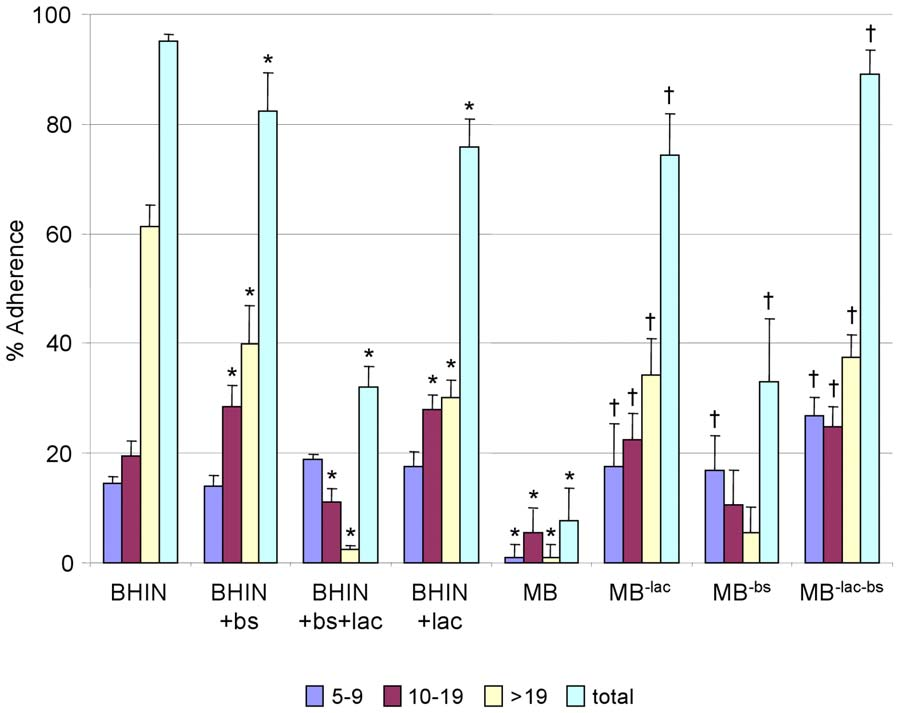
Effect of lactose and bile salts onadherence of EHEC O157:H7 strain 86-24 *in vitro*. Bacteria were grown in BHIN, BHIN+bs, BHIN+lac, BHIN+bs+lac, MB, MB^−lac^ (without lactose but containing bile salts), MB^−bs^ (without bile salts but containing lactose) and MB^−bs−lac^ (without bile salts and lactose) to IPEC-J2 cells. The bacterial strains were incubated with the cultured cells for 6 h and adherence was quantified by examining 100 cells for each assay and determining the mean percentage of cells (+SD) with bacterial clusters. 5–9: % of cells with a cluster of 5–9 adherent bacteria; 10–19: % of cells with a cluster of 10–19 adherent bacteria; >19: % of cells with a cluster of >19 adherent bacteria; Total: % of cells with a cluster of ≥5 adherent bacteria. * indicates *p*<0.05 as compared to BHIN; † indicates *p*<0.05 as compared to MB.

**Figure 2:**
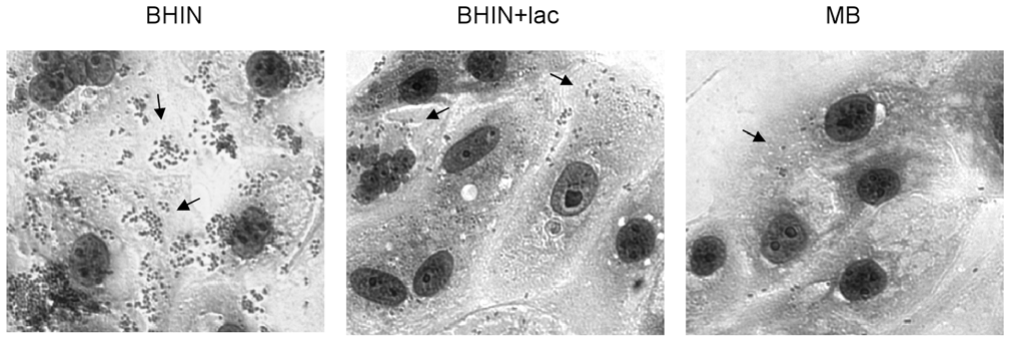
Illustration of adherence of EHEC O157:H7 strain 86-24 *in vitro* in the presence and absence of lactose. Bacteria were grown in BHIN, BHIN+lac, and MB to IPEC-J2 cells. Adherence assays were performed for 6 h as described in Materials and Methods. The bacteria grown in MB caused almost no adherence; there was a sharp decrease in adherence when grown with lactose.

In previous studies, there was very little adherence of bacteria cultured in MB to IPEC-J2 and HEp-2 cells [32]. The unique MB components, lactose and bile salts, were therefore examined for their effects on adherence. Addition of lactose to BHIN caused a marked reduction in total adherence and in large clusters on IPEC-J2 cells. Addition of bile salts to BHIN resulted in a slight reduction in adherence to these cells. When bile salts and lactose were both added, there was a further decrease in adherence (Figs. 1 and 2). To prove whether the effects by MB were due to other components in stead of lactose and bile salts, MB minus lactose and/or bile salts was examined in bacterial adherence to IPEC-J2 cells. Consistent with the effects by addition of lactose and bile salts to BHIN, this study showed that bacteria grown in MB minus bile salts (containing lactose) increased bacterial adherence, while MB minus lactose (containing bile salts) increased bacterial adherence more significantly, and MB minus both lactose and bile salts restored the adherence levels to those caused by bacteria grown in BHIN (Fig. 1). Similar effects were observed with HEp-2 cells (data not shown).

### Effects of lactose and bile salts on pH of the growth media

The pH was measured before and after growth of EHEC O157:H7 overnight. Addition of NaHCO_3_ to BHI slightly increased the pH to 7.7, however, after bacterial growth overnight, the pH dropped to 5.9 when lactose was included in the medium, and no pH change was observed with the addition of bile salts. The pH of MB culture dropped from 7.3 to 5.5 following overnight growth.

### Effect of cultures in MB, BHI, lactose and bile salts on expression of virulence-related genes *in vitro*

#### (1) Effects on house-keeping genes

Housekeeping genes are usually used as internal controls to normalize mRNA expression levels independent of growth conditions [38]. Expression profiles of five EHEC O157:H7 house keeping genes *gapA*, *mdh*, *rpoA* and *rfbA*, as well as 16SrRNA, for which universal primers were designed to target all bacterial 16SrRNA genes [39], were determined for the conditions tested. Expression of *gapA* and *rpoA* was increased significantly by lactose and bile salts while *mdh* was decreased by lactose and bile salts (Table 1). The fluctuation in expression of these genes that are involved in carbon metabolism likely represents a response to the differences in carbon source [30], [40]. Only *rfbA* for the *in vitro* and 16SrRNA for both *in vitro* and *in vivo* conditions were shown to be consistently expressed (Table 1). Gene *rfbA* is highly associated with *E. coli* O157:H7 and is responsible for the synthesis of O-antigen [41]. Results from both *rfbA* and 16SrRNA as reference genes were quite similar, and only the data from 16SrRNA as an internal control were shown. In the pig intestinal loops, amounts of rRNA from the normal microflora might not be uniform; however, data were obtained from the average of 4–6 loops to address the variability in individual loops.

**Table 1:** Quantification of transcripts (RFE) by qPCR for genes not included in the figures. a

Genes	BHIN	BHIN + bs	BHIN + lac	BHIN + lac + bs	MB	BHIN-Loop	MB-Loop	Control-Loop
*crr*	1±0.271	1.679±0.201	1.267±0.155	1.596±0.302	1.701±0.530	0.121±0.056 *	0.456±0.183 *†	0.018±0.008 *
*ptsG*	1±0.329	2.145±0.264 *	1.544±0.103 *	1.320±0.276	1.384±0.277	0.203±0.111 *	0.424±0.104 *	0.067±0.024 *
*himA*	1±0.272	1.057±0.149	0.711±0.204	0.975±0.151	0.896±0.370	0.113±0.037 *	0.419±0.358	0.037±0.006 *
*hns*	1±0.241	0.660±0.124	0.536±0.214	0.352±0.132 *	1.193±0.360	0.188±0.055 *	0.648±0.416 *	0.124±0.046 *
*hha*	1±0.362	1.481±0.398	0.579±0.214	0.55±0.206	1.063±0.564	0.245±0.172 *	0.316±0.179 *	0.056±0.025 *
*nleD*	1±0.259	3.365±0.435 *	0.975±0.135	1.383±0.144	1.129±0.426	0.189±0.102 *	0.397±0.257 *	0.007±0.005 *
*espJ*	1±0.645	2.329±0.916	1.823±1.367	0.909±0.627	1.528±0.447	0.192±0.091 *	0.262±0.15 *	0.015±0.014 *
*espP*	1±0.325	1.993±0.904	1.637±0.463	1.58±0.792	1.456±0.759	0.312±0.129 *	1.055±0.867	0.005±0.006 *
*espFu-TccP*	1±0.197	1.767±1.164	1.085±0.742	1.607±1.303	1.294±0.308	0.403±0.204 *	1.071±0.542	0.035±0.029 *
*chuA*	1±0.268	6.467±1.445 *	1.135±0.156	0.822±0.526	1.689±0.592	1.040±0.653	2.159±1.212	0.213±0.039 *
*gapA*	1±0.305	1.64±0.126 *	2.481±0.362 *	2.846±0.195 *	6.429±1.683 *	0.139±0.042 *	0.499±0.181 *	0.336±0.251 *
*rfbA*	1±0.217	1.313±0.448	1.351±0.294	0.937±0.271	1.398±0.333	0.297±0.214 *	0.518±0.141 *	0.007±0.005 *
*rpoA*	1±0.101	0.944±0.12 *	2.161±0.217 *	2.576±0.367 *	7.897±2.073 *	0.121±0.079 *	0.881±0.242	0.114±0.042 *
*mdh*	1±0.333	0.609±0.081	0.375±0.043 *	0.288±0.037 *	1.195±0.331	0.073±0.039 *	0.458±0.274	0.440±0.314
*16SrRNA*	1±0.052	1.037±0.057	1.048±0.073	1.098±0.065	1.102±0.055	1.014±0.04	0.993±0.051	1.126±0.106

a , Data are presented as relative fold expression (RFE) and represent the changes in transcription compared to the bacteria grown in BHIN (value of 1.0). RNAs were isolated from EHEC O157:H7 strain 86-24 grown in BHIN, BHIN+bs, BHIN+lac, BHIN+bs+lac and MB *in vitro* and the bacteria recovered from loops inoculated with bacteria grown in BHIN (BHIN-Loop) and MB (MB-Loop) as well as the control loops inoculated with EMEM (Control-Loop). The levels of 16SrRNA transcripts were used to normalize the Ct values. Data are expressed as the means ± SD for RNA extracted in 4–6 replicates.

*†, *P*<0.05 as compared to BHIN and to BHIN-Loop respectively.

#### (2) Effect on LEE gene expression

**Figure 3:**
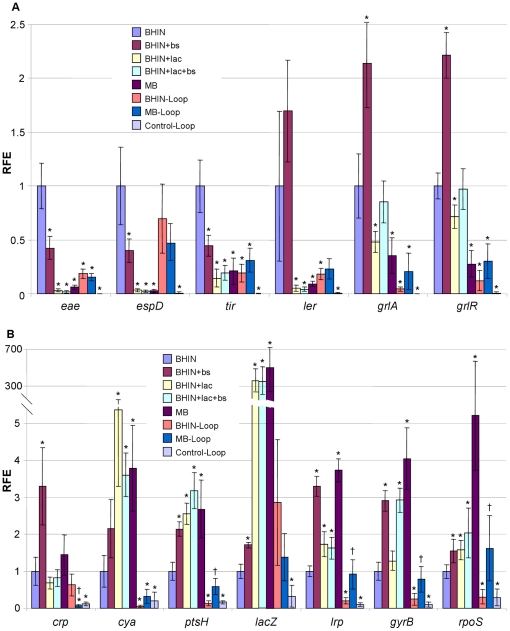
Transcriptional profiles of selected LEE, cAMP-CRP related and other regulatory genes. RNAs were isolated from EHEC O157:H7 strain 86-24 grown in BHIN, BHIN+bs, BHIN+lac, BHIN+bs+lac and MB at 37°C overnight statically, and the bacteria recovered from loops inoculated with bacteria grown in BHIN (BHIN-Loop) and MB (MB-Loop) at 37°C overnight statically as well as the control loops inoculated with EMEM (Control-Loop). Data are expressed as the means ± SD for RNA extracted in 4–6 biological replicates. Relative fold expression (RFE) represents the change in transcription compared to the bacteria grown in BHIN (value of 1.0). The levels of 16SrRNA transcripts were used to normalize the C_t_ values. * indicates *p*<0.05 as compared to BHIN; † indicates *p*<0.05 as compared to BHIN-Loop.

To investigate the underlying mechanisms of lactose and bile salts that affect adherence, a set of 68 genes including those encoding virulence and potential virulence factors and regulatory proteins were examined by quantitative PCR (qPCR) (Table S1). The qPCR analyses provided interesting insights into how lactose and/or bile salts affected the adherence phenotype as shown above. The intimate attachment related genes *eae*, *tir* and *espD* as well as the key regulator *ler* located in LEE1 were sharply decreased (>5-fold) by growth in lactose-containing media including MB (Fig. 3A). A 2-fold decrease for the positive regulator *grlA* by lactose and MB was also observed (Fig. 3A). Bile salts decreased *eae*, *tir* and *espD* gene transcripts only slightly and had no effect on *ler* expression; conversely, bile salts stimulated *grlA/R* expression (Fig. 3A).

#### (3) Effect on genes related to carbon catabolite repression (CCR)

To examine whether the effects of lactose were associated with the CCR effect, *cya* (encoding cAMP), *crp* (encoding cAMP receptor protein), *ptsH* (encoding HPr), *lrp*, *crr* (encoding EIIA), *ptsG* (encoding EIIBC), and *lacZ* (encoding β-galactosidase) were assessed. The results showed that culture in the presence of lactose increased *cya* transcripts 6.2 fold, and increased *ptsH*, *lrp* transcripts moderately (2.6 folds) (Fig. 3B). The genes *crp*, *crr* and *ptsG* were not affected by growth in lactose-containing media (Fig. 3B and Table 1). Bile salts had a large effect in stimulating *lrp* and *gyrB* expression (Fig. 3B). Gene *lrp* encodes leucine-responsive regulatory protein Lrp, a global transcription regulator [42]; and gene *gyrB* encodes DNA gyrase controlling DNA supercoiling and thus gene activities [43].

#### (4) Effects on acid-resistance genes

**Figure 4:**
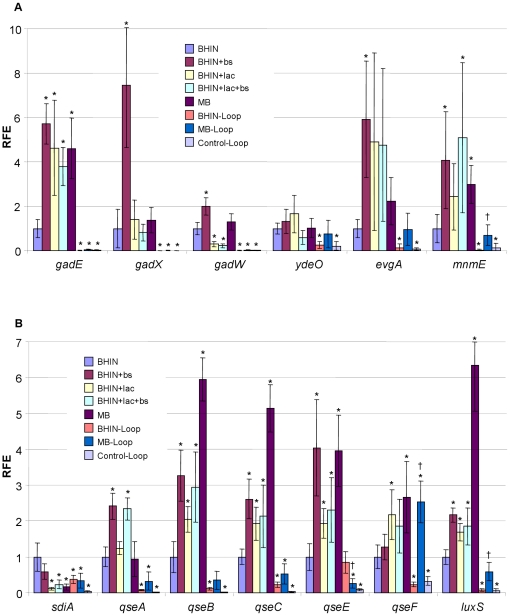
Transcriptional profiles of acid-resistance regulatory and quorum sensing genes. RNAs were isolated from EHEC O157:H7 strain 86-24 grown in BHIN, BHIN+bs, BHIN+lac, BHIN+bs+lac and MB at 37°C overnight statically, and the bacteria recovered from loops inoculated with bacteria grown in BHIN (BHIN-Loop) and MB (MB-Loop) at 37°C overnight statically as well as the control loops inoculated with EMEM (Control-Loop). Data are expressed as the means ± SD for RNA extracted in 4–6 biological replicates. Relative fold expression (RFE) represents the changes in transcription compared to the bacteria grown in BHIN (value of 1.0). The levels of 16SrRNA transcripts were used to normalize the C_t_ values. * indicates *p*<0.05 as compared to BHIN; † indicates *p*<0.05 as compared to BHIN-Loop.

Since growth in the presence of lactose reduced the pH, genes encoding the glutamate-dependent acid resistance (GDAR) system were examined. The key regulator of this system is GadE, which is also a negative regulator of the LEE. *gadE* was increased >3.8-fold by growth in the presence of lactose, bile salts and MB (Fig. 4A). *gadX*, *gadW*, *ydeO*, *evgA* and *mnmE*, the upstream regulators of *gadE*, were assessed. The data showed that *gadX*, *ydeO* and *evgA* were not changed by addition of lactose; on the other hand, addition of lactose decreased *gadW* expression (Fig. 4A). Bile salts stimulated the expression of *gadXW* and *evgA* significantly (Fig. 4A). *mnmE* was increased by MB, possibly via bile salts and/or mild low pH (Fig. 4A). *rpoS*, encoding general stress response factor sigma S, was slightly increased by lactose, and sharply increased by MB (5.2 fold) (Fig. 3B).

#### (5) Effects on quorum sensing and other regulatory genes

Lactose, bile salts and MB all stimulated gene expressions of *qseB*, *qseC*, *qseE*, and *luxS* (Fig. 4B). Lactose and MB but not bile salts also had a positive effect on *qseF*, while a 5-fold decrease for *sdiA* was observed for lactose and MB, the media with a low pH of 5.5 (Fig. 4B). An increase in *qseA* expression was only associated with the presence of bile salts (Fig. 4B). *luxS* transcripts were significantly increased by lactose and bile salts, and sharply increased (∼6.3 fold) by MB (Fig. 4B).

**Figure 5:**
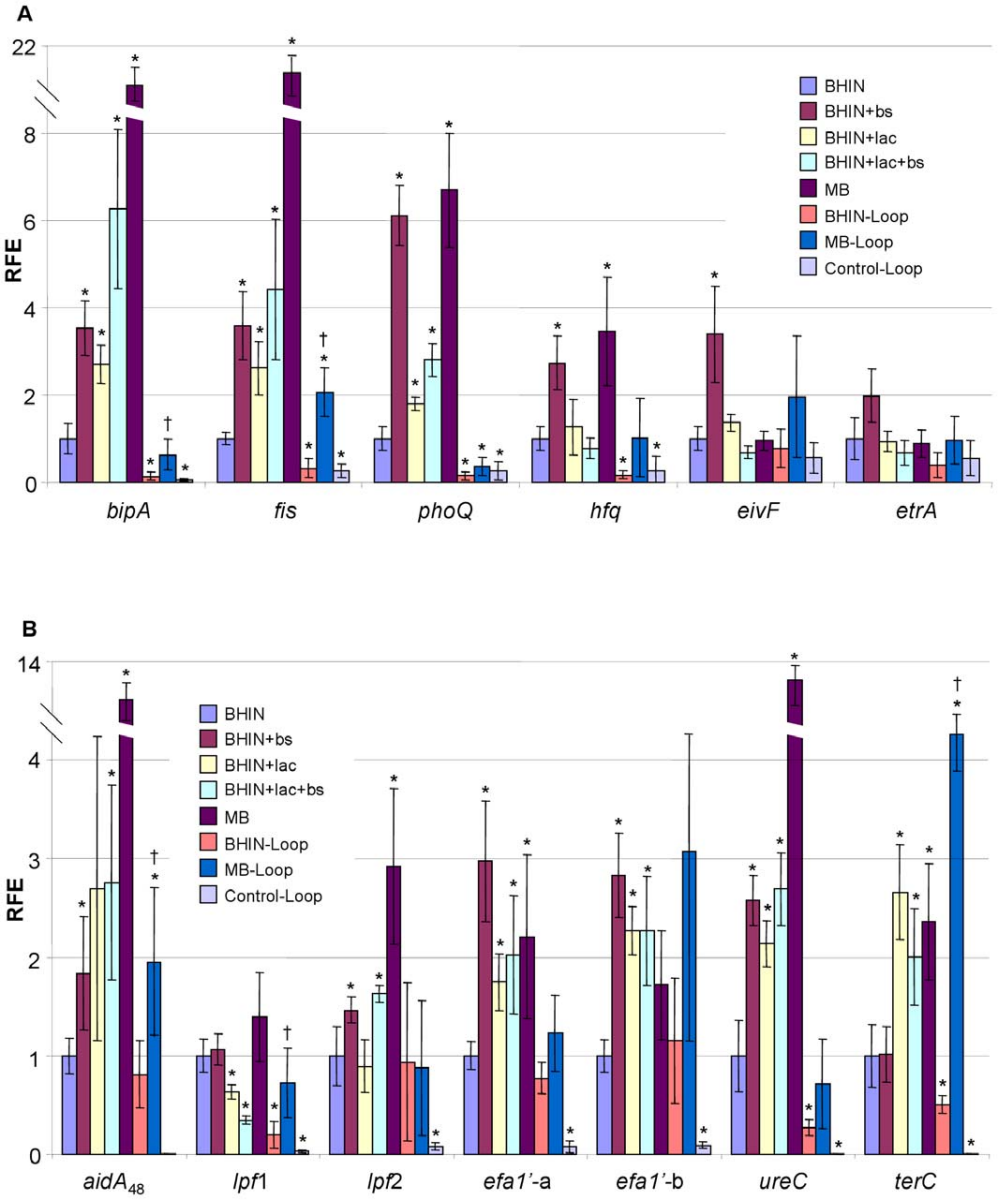
Transcriptional profiles of global and LEE regulatory and adherence related genes. RNAs were isolated from EHEC O157:H7 strain 86-24 grown in BHIN, BHIN+bs, BHIN+lac, BHIN+bs+lac and MB at 37°C overnight statically,and the bacteria recovered from loops inoculated with bacteria grown in BHIN (BHIN-Loop) and MB (MB-Loop) at 37°C overnight statically as well as the control loops inoculated with EMEM (Control-Loop). Data are expressed as the means ± SD for RNA extracted in 4–6 biological replicates. Relative fold expression (RFE) represents the changes in transcription compared to the bacteria grown in BHIN (value of 1.0). The levels of 16SrRNA transcripts were used to normalize the C_t_ values. * indicates *p*<0.05 as compared to BHIN; † indicates *p*<0.05 as compared to BHIN-Loop.

The global regulators *bipA* and *fis* were increased about 15.8 and 16.8 fold respectively by MB, while lactose and bile salts alone had a moderately positive effect (2.6- to 3.6-fold) (Fig. 5A). Lactose, bile salts and MB also increased expression of *phoQ* which encodes the sensor protein of the two-component phoQ/phoP system involved in magnesium acquisition (Fig. 5A). Other global regulators, *himA* (encoding IHF), *hns*, *hha*, *eivF* and *etrA*, were not affected by lactose, bile salts, or MB (Fig. 5A, Table 1). Interestingly, transcripts for the newly identified RNA chaperone protein Hfq [44], [45] were increased by bile salts and MB (∼2.7 and ∼3.5 fold, respectively). The larger effect of MB than bile salts indicated that the additive effect might also be attributed to the low pH (Fig. 5A).

#### (6) Effects on adhesins and potential adherence factors

**Figure 6:**
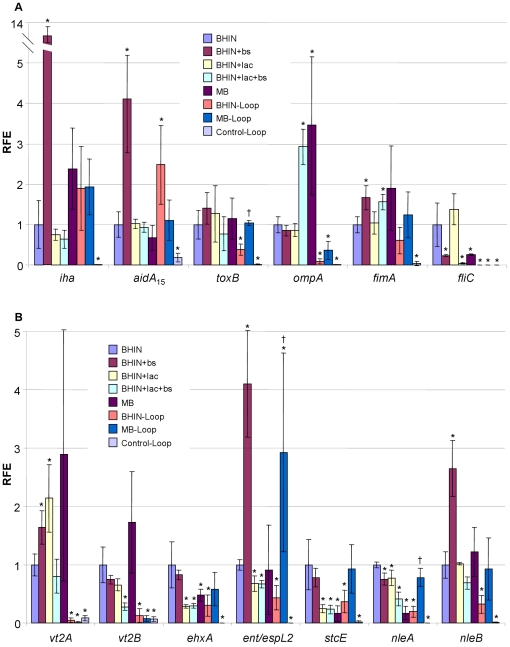
Transcriptional profiles of genes for putative adhesins, toxins, protease and secreted proteins. RNAs were isolated from EHEC O157:H7 strain 86-24 grown in BHIN, BHIN+bs, BHIN+lac, BHIN+bs+lac and MB at 37°C overnight statically and the bacteria recovered from loops inoculated with bacteria grown in BHIN (BHIN-Loop) and MB (MB-Loop) at 37°C overnight statically as well as the control loops inoculated with EMEM (Control-Loop). Data are expressed as the means ± SD for RNA extracted in 4-6 biological replicates. Relative fold expression (RFE) represents the changes in transcription compared to the bacteria grown in BHIN (value of 1.0). The levels of 16SrRNA transcripts were used to normalize the C_t_ values. * indicates *p*<0.05 as compared to BHIN; † indicates *p*<0.05 as compared to BHIN-Loop.

Genes encoding putative adhesins *aidA*_48_, *lpfA*_2_, *efa1*'*-*a, *efa1*'*-*b, and genes encoding the urease major subunit *ureC* and tellurite resistance *terC* were all significantly increased by MB, due to the effects of lactose and/or bile salts, notably, a substantial increase in *terC*, *aidA*_48_ and *ureC* transcripts was induced by MB (7.3, 9.2 and 11.3 fold respectively) (Figs. 5B). Lactose alone inhibited *lpfA*1 of OI-141, encoding long polar fimbriae, but this effect was not observed with MB (Fig. 5B). No significant effects on the genes encoding adhesins *aidA*_15_, *iha*, *toxB* and *fimA* (encoding pilin) were observed with MB, although bile salts alone remarkably activated *aidA*_15_ and *iha* expression (4.1 and 12.1 fold respectively) (Fig. 6A). It is worth noting that *ompA* was sharply increased by MB and the medium containing both lactose and bile salts; however, these effects were not seen with addition of lactose or bile salts alone (Fig. 6A). Bile salts caused a significant reduction in *fliC* expression (∼4-fold) (Fig. 6A).

#### (7) Effects on genes vt2, ehxA, ent/espL2, stcE and nleA/B

The gene *vt2A*, encoding the VT2 A-subunit, was significantly activated by lactose and bile salts, but this effect was not observed for *vt2B*, encoding the VT2 B-subunit (Fig. 6B). Both MB and lactose decreased putative virulence genes *ehxA*, *stcE* and *nleA* encoding EHEC hemolysin A, a metalloprotease and the non-LEE encoded gene A (NleA/EspI), respectively (Fig. 6B). NleA plays an important role in virulence of *C. rodentium* in mice [13], [46]. The genes *ent*/*espL2*(formerly *senA*) and *nleB* are both located in the OI-122, encoding secreted proteins which are delivered by the type III secretion system, and are associated with colonization of mice and virulence in humans [14], [47]. Both *ent/espL2* and *nleB* were activated significantly by bile salts alone (Fig. 6B).

### *In silico* search for CRP binding sites encoded by the LEE genes

Decreased LEE gene expression was concurrent with the cAMP increase in response to lactose. It is not known whether this effect was mediated by direct binding of cAMP-CRP near the promoter regions of the LEE genes. CRP binding sites contain a palindromic sequence in which two conserved motifs, TGTGA and TCACA, are separated by a spacer of ∼6 nucleotides [25]. A potential CRP binding site 5′-GATGAttttctTCTAT-3′ was found at -213 of the *ler* promoter region in LEE1. No potential CRP sites were identified by sequence analysis in the LEE5 and LEE4 promoter regions based on the promoter sequence data published [48], [49].

### Expression of virulence-related genes in bacteria from pig ligated intestine

#### (1) Inhibition test for the quantification of *in vivo* expressed genes

The genes *espD* and *tir* were efficiently amplified from the broth grown bacterial RNA mixed with RNA from the loop contents (data not shown). Amplification of *espD* and *tir* from the broth was also compared with amplification from the mixes of cDNA of the broth and the loops. Results showed that cDNA mixes produced the same levels of amplification as those of cDNA from the broth grown bacteria (data not shown), proving that the contents of the pig intestine contained no detectable inhibitory effect on cDNA synthesis and PCR reactions.

#### (2) Effects observed with bacteria cultured in MB and BHIN

Contrary to the poor adherence *in vitro*, bacteria cultured in MB adhered well to pig intestine [32]. Consistent with these observations, qPCR analysis revealed that in the intestine, the intimate adherence genes *eae* and *espD* and the regulators *ler* from bacteria grown in MB were expressed at significantly higher levels than those from cultures with lactose and MB *in vitro*, and notably, the expression of *espD* was close to that from BHIN culture under *in vitro* conditions (Fig. 3A). Gene *espD* is a member of the *espADB* polycistron that encodes the molecular syringe of the TTSS. Concurrently, the CCR related genes *cya* and *ptsH* were expressed at significantly lower levels in the intestine compared to those from the *in vitro* cultures, suggesting a change in carbon source in the intestine. Consistently, *lacZ* expression was significantly lower in the intestine than the media containing lactose, and similar to the *in vitro* media without lactose (Fig 3B). In addition, the *in vivo* transcript levels for *gadE*, *gadX* and *gadW* were almost nondetectable, and *phoQ* was also poorly expressed (Fig. 4A). The QS response regulator *qseF* and the global regulator *fis* were >2 fold higher in MB than in BHIN although the sensor kinase *qseE* was low (Figs. 4B and 5A). Other QS genes from MB and BHIN cultures were expressed at lower levels in the intestine (Fig. 4B). Furthermore, the negative regulators *hfq* and *hha* were expressed at a significantly lower level in the intestine than under *in vitro* conditions (Fig. 5A and Table 1). Growth in MB induced significantly higher expression of *lrp*, *gyrB* and *luxS* than did growth in BHIN in the intestine, similar to the trend in the *in vitro* conditions (Fig. 4B). Gene *rpoS* showed a similar trend of higher expression in MB than in BHIN culture *in vivo* (Fig. 4B).

A significant increase in the transcripts for the putative adherence related genes *aidA*_48_ (1.95-fold) and *terC* (7.3-fold) was observed with MB cultures in the loops compared with BHIN cultures in both *in vitro* and *in vivo* conditions (Figs. 5B and 6B). There was also a higher expression of the putative adhesin *toxB* with MB cultures compared with BHIN cultures in the loops (Figs. 6A). The putative adherence related genes *iha*, *aidA*_15_, *lpf2*, *fimA*, *efa1*'*-*a and *efa1*'*-*b from MB cultures were expressed at levels comparable to that from BHIN both *in vitro* and *in vivo* (Figs. 5B and 6A). BHIN caused a more pronounced effect on *aidA*_15_ transcripts, a significant ∼2.5-fold increase *in vivo* compared with *in vitro* (Fig. 6A).

In contrast to the trend *in vitro*, *nleA* was expressed at a significantly higher level from MB than from BHIN culture *in vivo*, and the pO157 encoded genes *ehxA* and *stcE* were expressed at high levels in the loops, similar to those in the *in-vitro* conditions (Fig. 6B). Remarkably, the putative toxin *ent/espL2* was increased significantly (2.93-fold) in the loops from MB culture than BHIN cultures in both *in vitro* and *in vivo* conditions (Fig. 6B). Expression of *espP* and *espFu*, encoding a serine protease from pO157 and Tir cytoskeleton coupling protein (TccP/EspFu) respectively, was relatively high in MB in the loops at levels similar to those observed in*-vitro* (Table 1).

Conversely, expression of *ompA* was >3-fold lower in the loops than *in vitro*; and *fliC* was almost non-detectable in the loops (Fig. 6A). Genes *vt2A* and *vt2B* from both MB and BHIN cultures were expressed >6 fold lower in the loops than the *in-vitro* conditions (Fig. 6B).

## Discussion

*In vitro* CCR was associated with down-regulation of genes involved in adherence of EHEC O157:H7 to epithelial cells. Decreased adherence of *E. coli* O157:H7 to cultured cells associated with growth in MB was mainly due to lactose; bile salts made a minor contribution. Analysis of transcriptional profiles by qPCR revealed that growth in the presence of lactose and in MB caused a decrease in LEE gene expression. Examination of CCR related genes showed that transcripts for *cya* (cAMP) were sharply increased by lactose/MB, which might cause the observed repression of *ler*, and consequently, the decrease in LEE gene expression. A potential CRP binding site was identified in the promoter region of *ler*. Transcripts for *stcE*, whose product promotes adherence, were also decreased, possibly due to the decrease in *ler* [17], [48], [49], [50],[51].

*In vitro*, transcripts for *gadE* were sharply increased by lactose and MB, which might contribute to repression of the LEE genes. GadE not only regulates the GDAR system of *E. coli* in response to acidity [52] but also represses expression of LEE genes, including *espB*, *espD*, *tir* and *ler* [53], [54]. Expression of *gadE* is controlled by multiple factors, including cAMP-CRP, RpoS, phoQ, MnmE [55], [56], [57]. Increased expression of these factors in the presence of lactose and MB could contribute to the activation of *gadE* expression. The upstream regulators GadX-W seemed not to be involved in the activation of *gadE* under these conditions. These effects could be due to both the reduced pH and CCR. It is not clear whether the increase in cAMP had a direct effect on *gadE* activation, or an indirect effect through *rpoS*, *phoQ* or *mnmE*. RpoS is a key regulator of acid resistance in EHEC and general stress response in *E. coli* [22], [58], and it negatively regulates LEE gene expression in EHEC strain EDL933 [59]. It is known that *rpoS* transcription is positively regulated by cAMP-CRP during energy starvation, stationary growth phase and stress conditions [50], [51], therefore, it is not possible to separate the effects of lactose and acidity with respect to the increase in cAMP and *gadE* expression.

The increase in *hfq* expression might also enhance the repression of LEE and non-LEE gene expression, and thereby contribute to the reduced adherence phenotype *in vitro*. It was recently reported that Hfq, possibly along with species-specific small RNA (sRNA), negatively regulates LEE- and non-LEE-encoded genes, especially *ler* [44], [45]. It is known that *hfq* is positively regulated by RpoS, while Hfq provides post-translational control of RpoS [60], [61].

Quorum sensing genes were upregulated although LEE gene expression was consistently repressed and a reduced adherence phenotype was observed. *qseB/C* and *qseE/F* were strongly activated, while *sdiA* was significantly repressed by lactose and MB. Since QS *qseB/C* and *qseE/F* positively regulate LEE gene transcription while QS *sdiA* negatively regulates LEE, activation of LEE genes and enhanced adherence might have been expected. It is possible that the repressive effects of cAMP, RpoS, GadE and Hfq overwhelmed the effects of *qseB/C*, *qseE/F* and *sdiA*. It cannot be ruled out that QS related genes might be subject to post-transcriptional regulation.

A decrease in the expression of *ehxA*, *stcE*, *ent* and *nleA* might also contribute to the decreased adherence phenotype. It is noteworthy that *aidA*_48_ and *terC* were highly expressed in cultures with lactose and in MB; the role of these genes in EHEC virulence is not clear. TerC was shown to be involved in adherence since a *terC* mutant caused a significant decrease in adherence to HEp-2 and IPEC-J2 cells by EHEC O157:H7 strain 86-24 [62].

The increase in expression of *cya*, *hfq*, *rpoS*, *qseB*/C and *qseE*/F by lactose and MB could be due to the dual effects of CCR and mild acidity (pH 5.5-5.9). Consistent with these observations, in separate experiments, prior exposure of EHEC O157:H7 strain 86-24 to pH 2.5 for 3-h and subsequent culture in BHIN overnight resulted in transcripts of *cya*, *hfq*, *rpoS*, and *qseF* that were increased ∼3, ∼3.2, ∼5.8, and ∼2.4-fold respectively (data not shown), suggesting that cAMP, Hfq and QS may be involved in bacterial acid-resistance.

**Figure 7:**
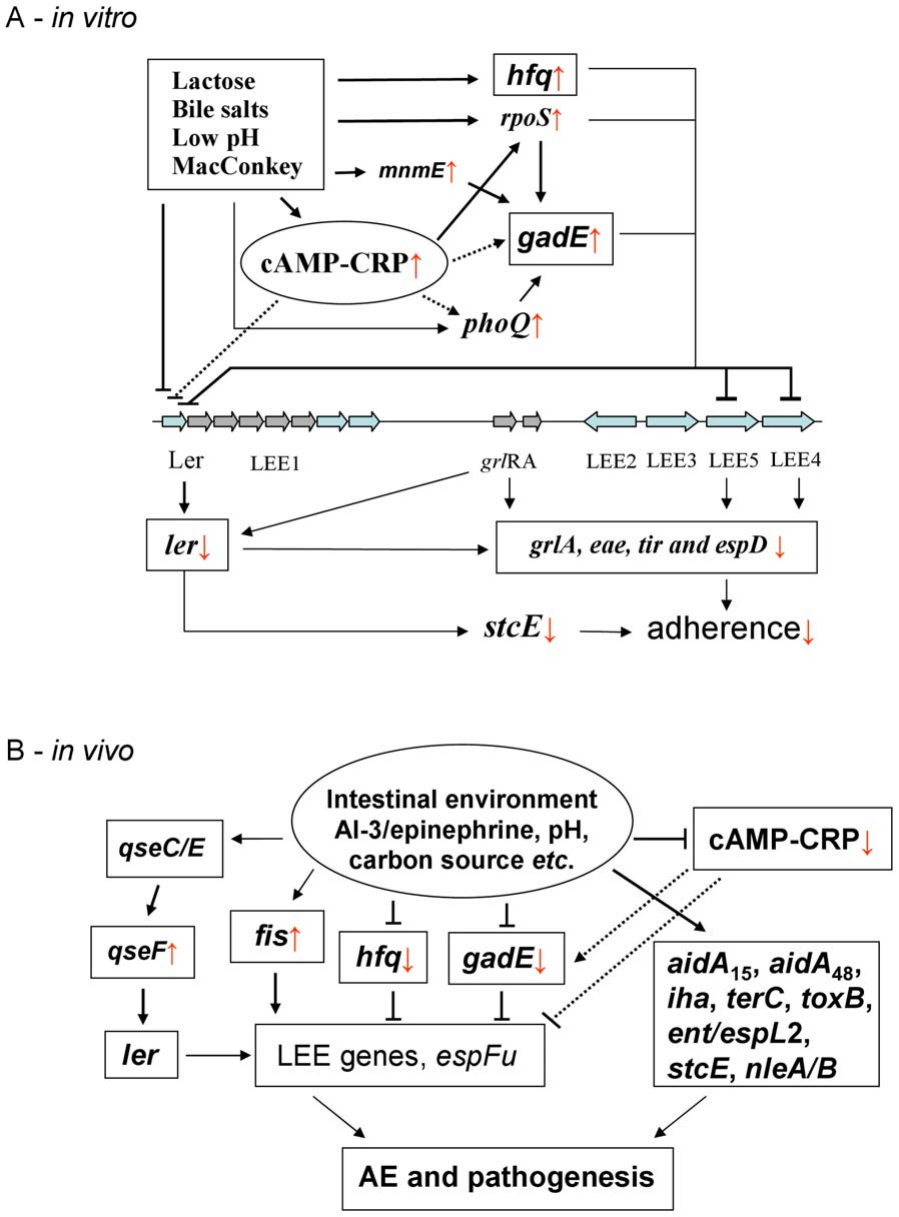
Schematic model of LEE gene regulation. Actions via cAMP-CRP, *hfq* and *gadE* in EHEC O157:H7 grown in lactose/bile salts, low pH and MacConkey *in vitro* (A), actions and the signalling cascade leading to AE and pathogenesis by the pathogen in the intestinal environment (B). The lines that end with arrowheads indicate positive effects; lines that end with crossbars indicate negative effects. Dashed lines indicate hypothetical interactions. The up- and down- arrows indicate up-regulated or down-regulated genes found in this study.

A model of the lactose effect via cAMP, GadE and Hfq in the repression of LEE genes and other adherence related genes is presented in Fig. 7, in which cAMP, GadE and Hfq may be major control factors in LEE gene expression. Obviously, prior culture conditions determined the adherence phenotypes, due to the underlying transcriptomic profiles as determined by the culture conditions. It seemed that, even during the 6 h of the *in vitro* adherence assay, these transcriptomic profiles were not restored by the assay medium and by contact with the cultured cells.

*In vivo*, LEE genes and other genes associated with adherence were highly expressed. The CCR related genes *cya* and *ptsH*, as well as *gadE* and *hfq* were expressed at low levels compared to the *in vitro* conditions, implying that the repressive effects by cAMP, GadE and Hfq were relieved in the intestine. As a result, LEE gene expression (especially *eae*, *espD* and *ler*) was relatively high. These changes explain the different adherence phenotypes *in vitro* and *in vivo*. It is interesting that *qseF* was markedly increased in loops compared to *in vitro*, and consequently, *espFu* was also expressed at a level comparable to that seen in BHIN *in vitro*. The expression of *espFu* could be activated by QseF [63]. These data suggest that QseF may provide important control of the expression of AE-related genes during EHEC infection. It is not clear what factors caused the higher expression of *qseF in vivo*. In the host intestine, AI-3/epinephrine/norepinephrine might stimulate *qseF* expression, because the AI-3/epinephrine and norepinephrine system controls the AE phenotype and flagellar motility through QseC sensor kinase [64], [65], [66]. Transcripts for *qseA* and *qseB/qseC* were uniformly low in the loops. Our data suggest that *fis* might also be involved in this regulation due to its high expression *in vivo* in MB culture.

The *in vitro* and *in vivo* assays differ in almost every aspect, including duration. However, each assay had as its end point adherence indicated by the positive controls. We sought to compare gene expression in association with adherence. It is highly unlikely that the differences in gene expression were simply due to the time differences for the two assay systems as we have observed that the 6 h *in vitro* assay involves early adherence which matures in 6 h. It is likely that the longer time needed in the pig loops is due to less opportunity for contact between the bacteria and the enterocytes compared with the *in vitro* system.

Gene transcripts for *aidA*_15_, *aidA*_48_, *terC* and *ent/espL2* were increased significantly *in vivo*, while putative adhesins *iha*, *efa1*'-a, *efa1*'-b, *toxB*, the non-LEE encoded genes *nleA* and *nleB*, and the pO157 plasmid encoded *ehxA* and *stcE* were expressed *in vivo* at levels comparable to the *in vitro* conditions. AidA_15_ was recently demonstrated to be involved in adherence by EHEC O157:H7 [9]. The high level of *espP* expression *in vivo* following MB culture supports the report that EspP influences the intestinal colonization of calves and adherence to bovine primary intestinal epithelial cells [67]. Together, these factors could all contribute to the *in vivo* adherence phenotype.

In summary, the present study showed that growth in lactose/MB inhibited LEE gene expression and concurrently increased *cya*, *gadE* and *hfq* expression associated with the decreased *in vitro* adherence phenotype of EHEC O157:H7. *In vivo*, expression of *cya*, *gadE* and *hfq* was low and was accompanied by a significant expression of LEE genes, which explains the observed AE lesions following inoculation of pig intestinal loops with bacteria grown in MB as described previously [32]. These data suggest that cAMP, GadE and Hfq may play major roles in the regulation of LEE gene expression. In the loops, *qseF* and *fis* expression was increased significantly, which might represent a response to AI-3/epinephrine or other intestinal cues, resulting in LEE gene expression and the AE phenotype. Significant expression of the putative factors *aidA*_15_, *aidA*_48,_ iha, *terC*, *toxB*, *ent*/*espL2*, *stcE*, *nleA*/B may indicate that these factors all contribute to adherence and pathogenesis by EHEC O157:H7 *in vivo*. Therefore, it is proposed that under acidic or standard laboratory growth conditions, expression of LEE and non-LEE-encoded effectors is silenced via the coordinated actions of cAMP, GadE and Hfq systems. When the pathogen encounters the host intestinal environment, changes in pH (∼ 6.93 in the ileum of pigs) and carbon sources cause a decrease in cAMP, GadE and Hfq and consequent de-repression of LEE and non-LEE effectors. Furthermore, factors like AI-3 produced by the intestinal microbiota and epinephrine produced by the host can activate LEE gene expression through QS. Activation of the non-LEE encoded factors such as *aidA*_15_, *aidA*_48,_ iha, *terC*, *toxB*, ent/*espL2*, *stcE*, and *nleA*/B also aid the infection process by the pathogen (Fig. 7).

## Materials and Methods

### Ethics statement

The experimental protocols and care of the animals were approved by the University of Guelph Animal Care Committee (Approval ID #05R143).

### Bacterial culture conditions, tissue cells and reagents

We used strain 86-24, a clinical isolate of EHEC O157:H7, for this study. HEp-2 (ATCC CCL23) cells were maintained in Eagle's Minimal Essential Medium (EMEM) (Invitrogen, Carlsbad, CA) and IPEC-J2 pig jejunal epithelial cells [32] were maintained in DMEM (Invitrogen). Both media were supplemented with 10% FBS, penicillin (100 IU/ml) and streptomycin (100 µg/ml). Brain-heart infusion (BHI) broth and MacConkey broth (MB) were purchased from Fisher Scientific (Nepean, Ontario, Canada). Lactose and bile salts were purchased from Sigma (St. Louis, MO).

### *In vitro* adherence assay

Bacteria were grown in 3 ml of medium in a 12-ml sterile plastic tube (Fisher Scientific) capped tightly and incubated statically overnight (16–18 h). The density of the cell cultures was adjusted photometrically so that cultures contained approx. 5×10^8^ cfu (colony forming unit)/ml prior to their use in the assay.

HEp-2 and IPEC-J2 cell adherence assays and quantification of bacterial adherence were conducted as described previously [32]. Briefly, approximately 2×10^5^ HEp-2 or IPEC-J2 cells per well were dispensed in 6-well cell culture plates (Corning, NY) and grown in EMEM or DMEM, respectively, overnight in the presence of 5% CO_2_. The cell monolayers at ∼ 50% confluency were washed and reconstituted with fresh EMEM (800 µl per well) without antibiotics. The cells were infected with a 20-µl volume of the overnight bacterial culture containing approximately 10^7^ cfu bacteria, that were grown in either BHI plus NaHCO3 (final concentration 44 mM) (BHIN), or BHIN plus lactose (1%) (BHIN+lac), or BHIN plus bile salts (0.5%) (BHIN+bs), or BHIN plus lactose and bile salts (BHIN+bs+lac), or MB, or MB-lactose (MB^−lac^, without lactose but containing bile salts), or MB-bile salts (MB^−bs^, without bile salts but containing lactose), or MB-lactose-bile salts (MB^−bs−lac^). After incubation for 6 h at 37°C in 5% CO_2_ with a medium change at 3 h, the plates were washed with PBS to remove unbound bacteria, fixed with 70% methanol, stained with 1∶40 Giemsa (Sigma), and examined by light microscopy. Adherence was quantified by examining 100 consecutive cells per well and recording the percentage of HEp-2 or IPEC-J2 cells with clusters of 5–9, 10–19, and >19 bacteria. The percentage of cells with at least 5 adherent bacteria per cell was calculated as a measure of total adherence. Data are expressed as the mean of at least three separate experiments ± standard deviation (SD).

### Pig gut-loop experiments

Bacteria were grown in either MB or BHIN at 37°C overnight statically, concentrated by centrifugation and resuspended in EMEM containing 10% fetal bovine serum (FBS) to prepare an inoculum of approximately 1×10^10^ or 5×10^10^ cfu/ml of bacteria.

A total of 63 12- to 14-day-old female pigs were used, with two or three pigs from the same litter being used at one time. The pig gut loop procedures were followed as described previously [32]. A 2-mL volume of inoculum containing either 2×10^10^ or 1×10^11^ cfu of the test organisms from cultures in either MB or BHIN respectively was injected into the lumen of the ileal loops through a 25 gauge needle. After inoculation, the ileum was replaced in the abdomen and the laparotomy incision was closed.

The pigs were euthanized by an overdose of pentobarbital 15–16 h after inoculation of the loops. The ligated segments of intestine were quickly excised and the loop contents were collected in RNAlater (Ambion, Texas) and treated at 4°C overnight for RNA isolation.

### RNA isolation

Bacteria were cultured overnight under the same conditions as those used for the *in vitro* adherence assays and were harvested by centrifugation and resuspended in 2 ml RNAlater. The suspensions were incubated at 4°C overnight for RNA isolation. Bacterial total RNA was isolated using the RiboPure™-Bacteria kit protocol (Ambion, Texas) with some modifications. Briefly, samples in RNAlater from both bacterial cultures and pig loop contents were mixed with equal volumes of PBS and centrifuged at 5000×*g* for 10 min. The bacterial pellets were resuspended in the lysis buffer, RNAwiz, transferred to 2 ml screw capped tubes containing ∼500 µl Zirconia beads and subject to bead-beating by a mini-Beadbeater (Bioscience products, Bartlesville, OK) for 90 sec twice with cooling on ice for 2 min in between. Subsequent steps for RNA isolation and purification and DNase I treatment were followed according to the manufacturer's protocol. The extracted RNA was treated several times with DNase I until it was confirmed to be free of genomic DNA contamination, as determined by PCR using RNA as the template. Total RNA was quantified by a NanoDrop ND-1000 spectrophotometer (NanoDrop Technologies, Wilmington, DE) and RNA quality was visualized by agarose gel electrophoresis.

### Reverse transcription (RT) and quantitative PCR (qPCR)

First-strand cDNA was synthesized from a 1.0 µg quantity of the DNase I treated bacterial total RNA using SuperScript II™-RT with 100 ng of random primer pd(N)9 according to the procedures recommended by the supplier (Invitrogen).

qPCR was performed using a Stratagene Mx3005p thermal cycler with iTaq™ SYBR® Green Supermix with ROX (BioRad, Hercules, CA). The cDNA was diluted 5-fold, and 1 µl of each diluted sample was added to a 25-µl reaction mixture, which contained 1x Supermix (containing SYBR Green I and ROX dyes, dNTPs, and 3 mM MgCl_2_), and 150 nM of each primer. The program was 4 min at 95°C, then 40 cycles of 95°C for 30 s, 53° to 59°C (depending on primers used) for 30 s, and 72°C for 30 s. On completion of amplification, a dissociation curve was made by holding the reaction at 95°C for 1 min and 41 cycles of heating by 1.0°C/cycle beginning at 55°C and ending at 95°C, with a duration of 30 s for each cycle. Fluorescence was measured after each annealing. The house-keeping gene for 16SrRNA was used as an internal control, with the primers specifically designed to amplify all bacterial mRNA universally [39], which is important for the normalization of RNA isolated from the contents of pig ligated loops containing normal microflora and the comparison of gene transcripts between *in vitro* and *in vivo* conditions. Relative mRNA levels of genes of interest were determined and were normalized to 16SrRNA using a modified 2^−ΔΔCt^ method [68]. qPCR data are expressed as the changes in expression levels compared to the levels of bacteria grown in BHIN.

### Inhibition test

To test whether the RNA isolated from the contents of pig intestine contained inhibitors of enzymatic reactions, RNA from bacteria grown in BHIN was mixed 1∶1 with RNA isolated from the contents of control loops, and the RNA mixtures were subject to cDNA synthesis and qPCR amplification for the unique sequences *espD* and *tir* from the LEE of EHEC O157:H7. To determine whether there was minor inhibitory effect of the RNA from the contents of the pig intestine on qPCR amplification, the levels of amplification for *espD* and *tir* were quantified by addition of cDNA from broth grown bacteria to the cDNA from the contents of control loops at ratios 1∶1 to 1∶5. Three biological samples were used for each of the RNA and cDNA mixes.

### Statistical analysis

All analyses were performed with SAS for Windows version 8.02 (SAS Institute Inc., Cary, N.C.). The *in vitro* adherence to cultured cells was compared by analysis of variance of the percentage adherence of clusters with 5–9, 10–19, and >19 bacteria per cell, as well as the total percentage adherence (≥5 adherent bacteria per cell) using PROC GLM. *P*-values ≤0.05 were considered significant.

## Supporting Information

Table S1 Primers used for quantitative PCR* and their target genes.
(DOCX)
