# Supplementary material for: Differential Gene Expression and Adherence of Escherichia coli O157:H7 In Vitro and in Ligated Pig Intestines
Source: PLoS One. 2011 Feb 28;6(2):e17424. doi: 10.1371/journal.pone.0017424 (PMC3046156; doi:10.1371/journal.pone.0017424)
Supplement: Table S1 — Primers used for quantitative PCR* and their target genes. (DOCX) [file pone.0017424.s001.docx]

Table S1. Primers used for quantitative PCR* and their target genes.

| **Primer** | **Nucleotide sequence (5' - 3')** | **Target gene** | **locus** | **product/function** |
| --- | --- | --- | --- | --- |
| **For LEE genes** | | | | |
| Tir-F | TTGTATTTACTGGAGGCCGTG | *tir* | Z5112 | Translocated intimin receptor |
| Tir-R | TCTGTGGTGTTTTCCGCAC |  |  |  |
| EspD-F | GAACGAATACCGTTCGTAACG | *espD* | Z5106 | Translocated protein |
| EspD-R | TTAAAGACTGCAGCACAGGTC |  |  |  |
| Eae-F | CTGGTGAAACTGTTGCCGATC | *eae* | Z5110 | Intimin |
| EaeR | CCACCTGCAGCAACAAGAGG |  |  |  |
| Ler-F | GGTCTGCCCTTCTTCATTGC | *ler* | Z5140 | Transcriptional regulator |
| Ler-R | AAATAATTCACATACAACAAGTCC |  |  |  |
| GrlA-F | ATCTCAAGGCGCTTATAATGC | *grlA* | Z5128 | Global regulator of LEE-activator |
| GrlA-R | TGTAAATTGCAGGAGAAATGG |  |  |  |
| GrlR-F | TAAACTTGTGGCATTCCTGTG | *grlR* | Z5129 | Global regulator of LEE-repressor |
| GrlR-R | CATCATACTTCATGTCCATCG |  |  |  |
| **For genes in carbon metabolism** | | | | |
| Crr-F | CCAACCACGCATTCTCTATC | *crr* | Z3683 | PTS system, glucose-specific EIIA |
| Crr-R | TTGGCTTTCTCTTCCAGCTG |  |  |  |
| PstG-F | CGCTTTGTGCCGATCATTTC | *ptsG* | Z1740 | PTS system, glucose-specific EIIBC |
| PstG-R | CGTTCCAGATGTGGTGCAG |  |  |  |
| Crp-F | CCAAGAGCACGCTTATTCAC | *crp* | Z4718 | Cyclic AMP receptor protein |
| Crp-R | TACCCATGCGCTACGTTCC |  |  |  |
| cAMP-F | GCTAATGCCGGGTTACCTTG | *cyaA* | Z5322 | Adenylate cyclase |
| cAMP-R | AGAGGAACAACTTTGCCCTAC |  |  |  |
| PtsH-F | TGTGACTTCCAACGGCAAAAG | *ptsH* | Z3681 | PTS system, histidine containing protein (HPr) |
| PtsH-R | TTCCGCCATCAGTTTAACCAG |  |  |  |
| LacZ-F | AAAGACCAGACCGTTCATGC | *lacZ* | Z0440 | β-D-galactosidase |
| LacZ-R | TTGGCGGCTTCGCTAAATAC |  |  |  |
| **For quorum sensing related genes** | | | | |
| SdiA-F | CTGTGCTTCGTTGAATAAGTC | *sdiA* | Z3004 | Quorum sensing, LuxR homologue |
| SdiA-R | CTGGAGTACGATTACTATTCG |  |  |  |
| QseA-F | AAGATGAGTTGCAGGTAAAGC | *qseA* | Z4602 | Quorum sensing *E. coli* regulator A |
| QseA-R | ACCAGATTGACGCTCAAACC |  |  |  |
| QseB-F | GATCCTGGATTTAACCTTACC | *qseB* | Z4377 | Quorum sensing *E. coli* regulator B, response regulator |
| QseB-R | GACTTCTATCAACGCAAAAGG |  |  |  |
| QseC-F | TTCGACACCCAACTGATGC | *qseC* | Z4378 | Quorum sensing *E. coli* regulator C, sensor kinase |
| QseC-R | ATCGCCATCGTTAAGGACC |  |  |  |
| QseE-F | GTACCATTACCAGACTCACC | *qseE* | Z3830 | Quorum sensing *E. coli* regulator E, sensor kinase |
| QseE-R | CTTCAGTGCAACAACAGTGG |  |  |  |
| QseF-F | TATCAACAGGCTTGGTGAGG | *qseF* | Z3833 | Quorum sensing *E. coli* regulator F, response regulator |
| QseF-R | TAGATTTAGTCATCAGCGACC |  |  |  |
| LuxS-F | CTTTCAGCACGTCTTCCATTG | *ygaG* | Z3988 | AI-2 synthase |
| LuxS-R | AAGAAGTGATGCCAGAAAGAG |  |  |  |
| **For global regulatory genes** | | | | |
| Hns-F | TTTACCTTGCTCATCCATTGC | *hns* | Z2103 | H-NS, histone-like protein |
| Hns-R | AATATCGCGAAATGCTGATCG |  |  |  |
| Hha-F | TCCATACTGAGGAAGGGATC | *hha* | Z0573 | Regulator, haemolysin expression modulating protein |
| Hha-R | GAAAAACCTTTAACGAAAACCG |  |  |  |
| HimA-F | TTTAACTTCTGCCCGGGTCTG | *himA* | Z2741 | Integration host factor alph subunit |
| HimA-R | GTTTTTCGAAGAGATCCGTCG |  |  |  |
| Lrp-F | TCTGGATGCATCACTTCTGG | *lrp* | Z1234 | Leucine-responsive protein |
| Lrp-R | GCAACTTACGGTAGGCTGAC |  |  |  |
| PhoQ-F | CGGTTCAGGTTTCGTACCAG | *phoQ* | Z1858 | Sensor protein PhoQ |
| PhoQ-R | TGTGCTCTCAGCCAATCTGC |  |  |  |
| GyrB-F | CATTGGTGAAGGTTTCGAGG | *gyrB* | Z5190 | DNA gyrase subunit B |
| GyrB-R | TTGGTGTTTCGGTAGTAAACG |  |  |  |
| EivF-F | CCGTAAAGCTCACCAAGATC | *eivF* | Z4198 | ETT2 encoded regulator |
| EivF-R | CTTTGCATCAGAAGTTGTGC |  |  |  |
| EtrA-F | TCAAACCAAATACAGAACCTG | *etrA* | Z4184 | ETT2 encoded regulator |
| EtrA-R | ATGAAACACATAACCGCAGG |  |  |  |
| BipA-F | CGTTCCAGATGCAGATTTC | *yihK* | Z5407 | GTP-binding factor |
| BipA-R | TTTCGATACGTTCCAGACC |  |  |  |
| Fis-F | CGAACAACGCGTAAATTCTG | *fis* | Z4621 | Factor for inversion stimulation |
| Fis-R | ATTGCATCACCATGTCCAAC |  |  |  |
| RpoS-F | AACCTGAATCTGGCGAACACG | *rpoS* | Z4049 | δ^S^ factor |
| RpoS-R | AGCGTTGCTGGACATCCTGG |  |  |  |
| GadE-F | ACTTGCCCCATAAGAATTCAC | *yhiE* | Z4925 | Transcription regulator, *yhiE* |
| GadE-R | CTCATACGTCTTAGTTTCAGC |  |  |  |
| GadX-F | ATTTCTGCATAGCGAAGATTC | *gadX* | Z4929 | Transcription regulator, *yhiX* |
| GadX-R | GTATCAAGGGATACGCTTTC |  |  |  |
| GadW-F | GGCAAACTGTCAGCTCATCG | *gadW* | Z4928 | Transcription regulator, *yhiW* |
| GadW-R | TGCTCGGTGATCCTCATTCG |  |  |  |
| EvgA-F | AGCGGGTGGAAACACTTAAG | *evgA* | Z3631 | Transcription regulator |
| EvgA-R | CGAAACCATTAGCGCCAGC |  |  |  |
| YdeO-F | TGACGGCGAGATCCTGATG | *ydeO* | Z2209 | Transcription regulator |
| YdeO-R | AGTCGGTACATTCGAAGCAC |  |  |  |
| MnmE-F | TGGAACTGCAGGGTCATGG | *mnmE* | Z5198 | TrmE, tRNA modification |
| MnmE-R | ATAAGATCGGCAATCGCCTC |  |  |  |
| Hfq-F | GCTGCAAGGGCAAATCGAG | *hfq* | Z5779 | Post-transcriptional regulator |
| Hfq-R | TATTCTGCGCGCTGCTACC |  |  |  |
| **For toxin related and protease genes** | | | | |
| VT2A-F | ATACCACTCTGCAACGTGTCG | *vt2A* | Z1464 | Vero toxin, A subunit |
| VT2A-R32 | GTATACACAGGAGCAGTTTCAG |  |  |  |
| VT2B-F | G GTTGACGGGAAAGAATAC | *vt2B* | Z1465 | Vero toxin, B subunit |
| VT2B-R03 | AAACTGCACTTCAGCAAATCCG |  |  |  |
| EhxA-F | AGCTGCAAGTGCGGGTCTG | *ehxA* | L7048/pO157 | EHEC hemolysin |
| EhxA-R | TATTGTTGTCAGGGCTGCATC |  |  |  |
| StcE-F | CTCGGTGGGTAACACTCTTC | *stcE* | L7031/pO157 | Metalloprotease |
| StcE-R | TCATTTAGGCGACCGCTCAG |  |  |  |
| SenA-F | AATTCAGGAAAGATAGACTACG | *ent/espL2* | Z4326 | Effector protein |
| SenA-R | TGAACTTCTAATGATGTGTGAG |  |  |  |
| pagC-F | TTACAGACATATTGCCCCACG | *pagC* | Z4321 | PagC-like toxin |
| pagC-R | CCAGCTTATCGTCTGAATGAC |  |  |  |
| EspJ-F | ATTCTCTGACCAAAATGGAGC | *espJ* | Z3071 | *E. coli* secreted protein J |
| EspJ-R | GATTGTTTTGGATTCTTACAGC |  |  |  |
| EspP-F | ACCATTTCCTGACTTCTCACC | *espP* | L7020/pO157 | Serine protease |
| EspP-R | CGGGTCTCAACAACAAACTTG |  |  |  |
| **For adherence related and fitness genes** | | | | |
| Tccp-F | CGCAACAACTCTCTTTCGAG | *espFu* | Z3072 | EspFu, Tir cytoskeleton coupling protein |
| TccP-R | TGCCTCACATTAGGAAGTTG |  |  |  |
| Iha-F | GCGGAGGCTCTGAGATCAGT | *iha* | Z1617 | IrgA homologue adhesin |
| Iha-R | TGAACCCGGTATTCATGGCAG |  |  |  |
| 48aidA-F | GTGATACGGTTATTCGTGACG | *aidA_48_* | OI-48 | AIDA-like protein |
| 48aidA-R | TATACGAGTTGTCAGAATCAGG |  |  |  |
| 15aidA-F | GGTGAAGTTAATGCCAGGAAG | *aidA_15_* | Z0402 | AIDA-like protein |
| 15aidA-R | TGTCTTTGCCCACACAAACTC |  |  |  |
| OmpA-F | AAGTGCTTGGTCTGTACTTCC | *ompA* | Z1307 | Outer membrance protein A |
| OmpA-R | GGTGTTGAGTACGCGATCAC |  |  |  |
| NleA-F | TTGCATCGACTGGAACATCAG | *nleA* | Z6024 | Non-LEE encoded effector A |
| NleA -R | CCTCCGTGTAATTTGCTTTCG |  |  |  |
| NleB-F | GGAATTTGGCCATGTTGAAGG | *nleB* | Z4328 | Non-LEE encoded effector B |
| NleB-R | ATCTACATGCACAGCGATACC |  |  |  |
| NleD-F | ATCAGCAGATTGAAGCAGCAC | *nleD* | Z0990 | Non-LEE encoded effector D |
| NleD-R | AGTGAAAATCGGAACCAGTCC |  |  |  |
| LpfA141-F | CATCACCAACACCACTAAAGC | *lpfA1* | Z4965 | Putative fimbrial subunit |
| LpfA141-R | ATTTACAGGCGAGATCGTGGA |  |  |  |
| LpfA154-F | AGGTTTCCGGGCATTGAGTC | *lpfA2* | Z5225 | Putative major fimbrial subuit |
| LpfA154-R | TATGGCAGGTCACCTACAGG |  |  |  |
| Efa1-AF | GGATAAAAACAGTCGTAATGG | *efa1'-a* | Z4332 | Potential adhesion, ORF-A |
| Efa1-AR | TCTTAATATAGTCCGAGATGC |  |  |  |
| Efa1-BF | ACTGAAATGTTGCTTCGATGG | *efa1'-b* | Z4333 | Potential adhesion, ORF-B |
| Efa1-BR | TACACCATCAGATATTGCACG |  |  |  |
| FliC-F | CGAGATTTGATTTCGTCCTGG | *fliC* | Z3013 | Flagellin |
| FliC-R | TCTAACATTAAAGGCCTGACTC |  |  |  |
| ToxB-F | TCTGGTACAATTCCGTAATGG | *toxB* | L7095/pO157 | Potential adhesin/putative cytotoxin |
| ToxB-R | TAACCATCATCATCTAACATCG |  |  |  |
| Pilin-F | CAGCTGAATGATTGCGATACC | *fimA* | Z5912 | Major type 1 subunit fimbrin |
| Pilin-R | TTGTTTGCTCACTGAATGTCG |  |  |  |
| TerC-F | GGATTACTCCCAGCATCTGG | *terC* | Z1613 | Tellurite resistance protein subunit C |
| TerC-R | GTCCGAGAGTTCAACCACAG |  |  |  |
| UreC-F | CATTAACCCACGGTATTGCTC | *ureC* | Z1584 | Urease subunit C |
| UreC-R | CGAACATTGGGCGATAGTGC |  |  |  |
| ChuA-F | GGCTGAATCAATTTGCCAGG | *chuA* | Z4911 | Iron uptake system |
| ChuA-R | TCTTTGGTACTGGCGGCAC |  |  |  |
| **For house keeping genes** | | | | |
| RfbA-F | CGATGCAATTGGTACATTACG | *rfbA* | Z3198 | GDP-mannose dehydratase |
| RfbA-R | CTCGATAATTTACCGTGATCC |  |  |  |
| Mdh-F | AAGAAACGGGCGTACTGACC | *mdh* | Z4595 | Malate dehydrogenase |
| Mdh-R | GTGGCTGATCTGACCAAACG |  |  |  |
| RpoA-F | TCAGGTTGAGCAGGATTTCC | *rpoA* | Z4665 | RNA polymerase, alpha subunit |
| RpoA-R | TGACCCTTGAGCCTTTAGAG |  |  |  |
| GapA-F | CCGTTGAAGTGAAAGACGGTC | *gapA* | Z2818 | Glyceraldehyde-3-phosphate dehydrogenase A |
| GapA-R | AACCACTTTCTTCGCACCAGC |  |  |  |
| HDA-1F | ACTCCTACGGGAGGCAGCAG | *16SrDNA* | V3 region | 16SrRNA |
| HDA-2R | GTATTACCGCGGCTGCTGGCAC |  |  |  |

*All the primers were designed in the present study unless referenced.
